# Feasibility of improving identification of familial hypercholesterolaemia in general practice: intervention development study

**DOI:** 10.1136/bmjopen-2016-011734

**Published:** 2016-05-26

**Authors:** Nadeem Qureshi, Stephen Weng, Jennifer Tranter, Alia El-Kadiki, Joe Kai

**Affiliations:** 1 Division of Primary Care, NIHR School of Primary Care Research, University of Nottingham, Nottingham, UK; 2 Nottingham University Hospitals NHS Trust, Nottingham, UK

**Keywords:** PRIMARY CARE, GENETICS

## Abstract

**Objectives:**

To assess the feasibility of improving identification of familial hypercholesterolaemia (FH) in primary care, and of collecting outcome measures to inform a future trial.

**Design:**

Feasibility intervention study.

**Setting:**

6 general practices (GPs) in central England.

**Participants:**

831 eligible patients with elevated cholesterol >7.5 mmol/L were identified, by search of electronic health records, for recruitment to the intervention.

**Intervention:**

Educational session in practice; use of opportunistic computer reminders in consultations or universal postal invitation over 6 months to eligible patients invited to complete a family history questionnaire. Those fulfilling the Simon-Broome criteria for possible FH were invited for GP assessment and referred for specialist definitive diagnosis.

**Outcome measures:**

Rates of recruitment of eligible patients, identification of patients with possible FH, referral to specialist care, diagnosis of confirmed FH in specialist care; and feasibility of collecting relevant outcome measures for a future trial.

**Results:**

Of 173 general practices, 18 were interested in participating and 6 were recruited. From 831 eligible patients, 127 (15.3%) were recruited and completed family history questionnaires: 86 (10.7%) through postal invitation and 41 (4.9%) opportunistically. Among the 127 patients, 32 (25.6%) had a possible diagnosis of FH in primary care. Within 6 months of completing recruitment, 7 patients had had specialist assessment confirming 2 patients with definite FH (28.6%), and 5 patients with possible FH (71.4%). Potential trial outcome measures for lipid tests, statin prescribing and secondary causes of hypercholesterolaemia were extracted using automated data extraction from electronic records alone without recourse to other methods.

**Conclusions:**

The intervention is feasible to implement in GP, and facilitates recruitment of patients with raised cholesterol for targeted assessment and identification of FH. Extracting data directly from electronic records could be used to evaluate relevant outcome measures in a future trial.

**Strengths and limitations of this study**

- This feasibility study was able to engage general practices (GPs) and patients from underserved populations in an intervention to identify familial hypercholesterolaemia (FH) more systematically.
- Extraction of data using automated GP computer searches can capture important outcome measures for a future trial of FH identification.
- Further strategies are needed if engaging eligible patients is to be improved on opportunistic contact during GP consultations.
- The 6-month patient follow-up period used was too short to elicit complete data on all relevant outcome measures, such as eventual specialist assessment.

## Introduction

Familial hypercholesterolaemia (FH) is one of the most common inherited autosomal dominant disorders and is associated with elevated low-density lipoprotein cholesterol levels. In the UK, around 1 in 500 to 1 in 200 people are affected by the heterozygote form of this condition.^1^ Left untreated this can lead to premature coronary heart disease in those individuals affected.^2^ ^3^ However, with appropriate lipid-lowering treatment, intervention is highly effective and life expectancy can return to normal.^4^

Despite the overwhelming case for treatment and national guidelines recommending early identification, it is estimated that up to 80% of heterozygote FH still remain unrecognised.^5^ ^6^ Of most concern, individuals with raised cholesterol levels documented in general practice (GP) medical records may not be recognised to have possible FH. However, primary care is an ideal setting to identify possible FH cases through identification of those with raised cholesterol and relevant family histories. Current UK National Institute for Health and Care Excellence (NICE) guidelines recommend that these patients are identified using Simon-Broome criteria (box 1).^6^ ^7^

Box 1 **Referral criteria for diagnosis: abridged Simon-Broome diagnostic criteria**

- In adults, total cholesterol >7.5 mmol/L and low density lipoprotein >4.9 mmol/L.
- Plus for a diagnosis of possible familial hypercholesterolaemia (FH), family history of myocardial infarction at age <60 years in first-degree relative, age <50 years in second-degree relative, or a family history of raised cholesterol levels.
- Plus for a diagnosis of definite familial hypercholesterolaemia, tendon xanthomata in patient or in first-degree or second-degree relative.
- Patients with ‘definite’ or ‘possible’ familial hypercholesterolaemia should be referred to a specialist with expertise in familial hypercholesterolaemia, to confirm the diagnosis, management and coordination of the testing of relatives.

Using these criteria, a positive family history is based on patients recalling premature coronary heart disease or raised cholesterol levels in their first-degree and/or second-degree relatives. This level of detail is absent or poorly recorded in GP electronic health records (EHRs),^8^ so further work to collate the family history is required, such as using a validated self-administered family history questionnaire.^9–12^ Using a combination of searching GP EHRs for patients with raised cholesterol, with completion of a family history questionnaire, individuals with possible FH may be more actively and appropriately identified in primary care.

In line with Medical Research Council guidelines,^13^ this feasibility study aimed to inform the development of an intervention to identify FH more proactively in primary care. We investigated if the approaches used, study procedures and analysis were feasible, or might be enhanced, prior to their use in a future randomised controlled trial (RCT).^13^ We explored patients being targeted and invited for further assessment of possible FH, opportunistically when they consult, using computerised patient-specific reminders when their serum cholesterol is above diagnostic threshold (7.5 mmol/L) and was recorded in electronic medical records.^14^ ^15^ In addition, we explored an approach of practices simply mailing all patients with a cholesterol level ever recorded above diagnostic threshold. The specific objectives were to gain experience of:

- patient recruitment, identification, referral and diagnosis rates
- using opportunistic and universal postal recruitment strategies
- whether relevant outcome measures for a proposed trial may be extracted from automated searches of GP EHRs.

## Methods

### Study design

This was a feasibility study with process evaluation to inform a RCT. The study duration was 17 months, ending in August 2015, and was approved by NRES Committee West Midlands—Solihull (Reference 12/WM/0322).

### Participants

All 173 general practices in Nottinghamshire were invited to participate in the study through the local Primary Care Clinical Research Network, and we received expressions of interest from 18 practices. Further information was given to all 18 practices, and the first six eligible practices which responded, were recruited to the study (4 inner city practices, 1 suburban and 1 rural practice).

Eligible patients were aged 18 years or over at the start of the study in the recruited general practices with a previous record, in the computer medical records, of cholesterol at a level >7.5 mmol/L. Those who already had a confirmed diagnosis of familial hypercholesterolaemia were excluded.

### Intervention and procedure

The intervention began with a one-hour educational session at each recruited practice. This involved an update session on the identification and investigation of FH in line with current NICE guidelines,^7^ and linked to Simon-Broome criteria.^6^ General practitioners were familiarised with the proposed computer-based alert message which appears when eligible patients' medical records are accessed. They were also given a laminated sheet to use when eligible patients consulted their general practitioners as an aide memoire for further assessment. This outlined the Simon-Broome criteria, and a prompt to investigate and exclude secondary causes of hypercholesterolaemia such as diabetes, hypothyroidism, chronic kidney disease and liver disease.

Following training, a baseline computer data extraction was completed which identified 831 eligible patients, with cholesterol levels above 7.5 mmol/L from the six practices. These patients' EHRs were tagged with an alert message to check for secondary causes of elevated cholesterol, invite the patient to participate in the study, repeat the lipid profile and manage in line with NICE guidelines/Simon-Broome criteria for FH.^7^ This alert appeared when the individual's computer records were accessed. This opportunistic approach was continued for 6 months. Four months into the intervention, eligible patients identified at baseline who had not already been invited opportunistically, were all approached through a postal invitation from their practice.

Eligible patients were given study packs by their general practitioners opportunistically, or had them mailed to them by their practice. These included study information/consent and blood test forms to have a follow-up fasting lipid profile (comprising total cholesterol, LDL cholesterol and triglyceride) unless lipid profile was done in the last 6 months, a Family History and Symptom Questionnaire (FHSQ) seeking details on family history of myocardial infarction and cholesterol, together with photos to identify tendon xanthoma in Achilles tendons and hands. The consent form and FHSQ were returned to the research team office, with recruited patients' participation in the study using a computer recruitment code tagged onto the patient's GP EHRs. The research team then collated information on fasting lipid results, current stain therapy, family history and reported any possibility of FH diagnosis, in line with Simon-Broome criteria (box 1) back to GPs to facilitate contacting participants. Participants fulfilling Simon-Broome criteria for possible FH were asked to arrange an appointment with their general practitioner. The general practitioners were advised to examine patients for signs of FH, exclude secondary causes of raised cholesterol, and refer those patients who met Simon-Broome criteria to the local lipid specialist clinic. Those not fulfilling FH diagnosis based on Simon-Broome criteria were reassured, and a healthy living leaflet provided.

### Outcome measurements

The primary measures assessed the feasibility of this intervention (see box 2). Recruitment rate was determined during the 6 month study period, while referral, diagnosis and retention rate were assessed up to 6 months after completing recruitment (12 months after the start of the intervention). Diagnosis of confirmed FH in secondary care was based on clinical criteria (ie, Simon-Broome criteria). Relevant outcome measures for a proposed follow-up RCT were derived from the primary care recommendations in the NICE FH guidelines. This includes repeat testing for full lipid profile; excluding secondary causes of raised cholesterol and collecting comprehensive family history. The feasibility of extracting such data from GP EGRs was assessed using both an automated process via medical codes and a manual process in which a researcher reviewed patients' records, including free text entries and hospital letters. This was performed 6 months after the start of the intervention in each practice.

Box 2 **FAMCHOL feasibility study outcome measures assessed**

Primary measures

- Recruitment rate;
- Referral rate;
- Diagnosis rate:
  Possible familial hypercholesterolaemia (FH) identified primary care,
  Possible FH confirmed by secondary care,
  Definite FH confirmed by secondary care;
- Retention rate;

Extracting relevant outcome measures for proposed trial

- Cholesterol level:
  Latest total cholesterol levels (mmol/L),
  Latest low density lipoprotein (LDL) cholesterol levels (mmol/L),
  Proportion of patients who have a total cholesterol ≥7.5 mmol/L or LDL-C ≥4.9 mmol/L;
- Repeat cholesterol (lipid profile) test;
- Patients diagnosed with secondary cause (diabetes, hypothyroidism, chronic kidney disease, liver disease);
- Family history of coronary heart disease assessed;
- Quality of coronary heart disease family history assessed (age, condition, degree of relation);
- Thyroid stimulating hormone assessment (proxy for hypothyroidism investigations);
- HbA1c assessment (proxy for diabetes investigation);
- Serum creatinine assessment (proxy for chronic kidney disease investigations);
- Bilirubin, alkaline phosphatase (ALP) or glutamyl transpeptidase (gamma GT) assessment (proxy for liver disease);
- Arcus senilis or xanthalasma diagnosed;
- Proportion of patients prescribed statins;
- Proportion of patients prescribed high potency statins treatment (simvastatin ≥80 mg/day; atorvastatin ≥20 mg/day; rosuvastatin ≥10 mg/day)
- Dietary or weight management advice;
- Smoking cessation advice.

### Statistical analysis

As a feasibility study, we assessed the feasibility of the intervention, recruitment process and study instruments; hence, a sample size for proposed outcome measure was not appropriate. All primary outcome measures (recruitment, referral, retention and diagnosis rates) were presented descriptively. For proposed trial outcome measures (NICE guideline compliance), a descriptive analysis presented numbers and percentages for all categorical variables; mean and SD for all continuous variables at 6-month follow-up. All analyses were conducted in STATA 14 MP4.

## Results

### Primary outcomes: feasibility of FAMCHOL study process

#### Recruitment rate

**Figure 1:**
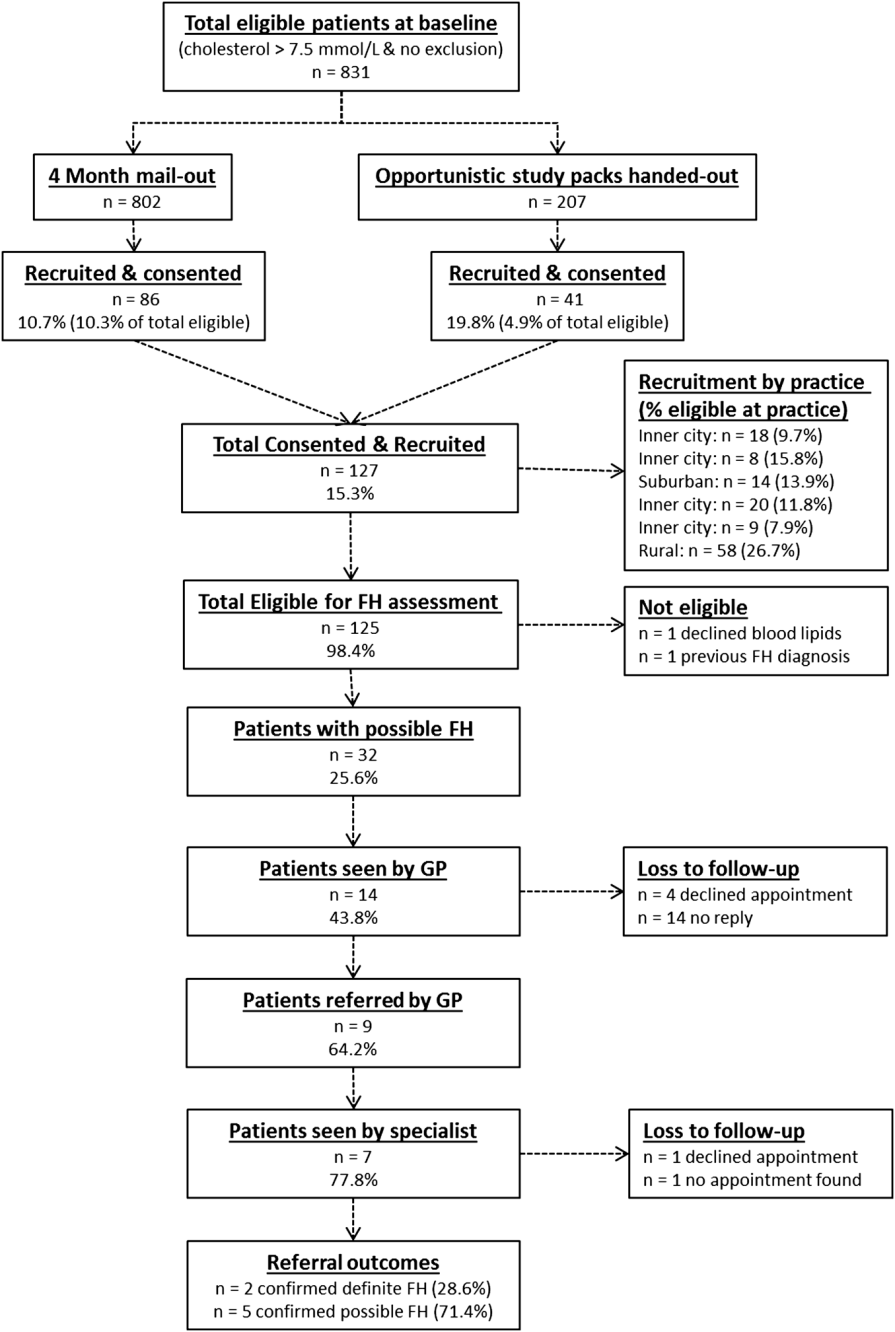
FAMCHOL study procedure and process. FH, familial hypercholesterolaemia; GP, general practice.

Figure 1 outlines the study recruitment process and procedure for the 831 eligible patients (2.36% of total) identified at baseline from 35 438 patients, over 18 years, registered with the six GPs.

Opportunistically, out of 207 study packs handed out, 41 (19.8% of study packs; 4.9% of all eligible patients) completed consent forms and Family History/Symptom Questionnaire. At 4 months, 802 study packs were mailed out to the outstanding eligible participants, and an additional 86 (10.7% all mail-outs; 10.3% of all eligible patients) were recruited using this systematic approach. By the end of the 6-month recruitment process, 127 patients consented and were recruited to the study which gave an overall response and engagement rate of 15.3%, (ranging from 7.9% to 15.8% at the four inner city practices, 13.9% at the suburban practice and 26.7% at the rural practice).

#### Identification and referral rate

Among the 127 consenting patients, 125 could be assessed for FH against Simon-Broome criteria. One patient declined follow-up blood lipids, and another patient had previously been diagnosed with familial hypercholesterolaemia on more detailed review of previous medical records. Thirty-two (25.6% of 125 eligible for assessment) patients had a possible diagnosis of FH and were invited to see their general practitioners. Fourteen patients (43.8% of 32 with possible FH) were actually seen by their general practitioner, while four patients declined the appointment and 14 patients did not respond to the invitation. Subsequently, nine patients (64.2% of 14 seen by GPs) were referred to lipid specialist. Further, seven patients (77.8% of the nine referred by GPs) were actually seen by specialists, as one patient declined the referral appointment and the other patient's referral could not be found in secondary care by the 12-month study period end.

#### Diagnosis rate

Of the seven patients with probable FH referred and seen by a specialist, two patients (28.6%) were confirmed with definite FH (by specialist examination) and five patients (71.4%) were confirmed with possible FH (mutation-negative for monogenic FH).

### Proposed trial outcomes: feasibility of data extraction

From 127 participants recruited, data on clinical records from 118 (95%) patients could be extracted from GP EHRs. Data from seven patients were excluded from the analysis due to transferring from practice. Table 1 details patient clinical characteristics based on the proposed outcome measures for 118 patients at 6 months from the start of the intervention through automated extraction from GP EHRs. Although all patients had a cholesterol assessment completed, 3% (n=4) of the sample did not have the LDL cholesterol measurement documented into their EHRs.

Among the 118 recruited patients, there were more men than women (61% men, 39% women), with an average age being 58 years (SD 9.0) for men and 56 years (SD 7.5) for women. The latest mean total cholesterol was 5.8 mmol/L (SD 1.5) and 6.4 mmol/L (SD 1.4) for men and women, respectively. The mean LDL cholesterol was 3.6 mmol/L (SD 1.3) and 3.8 mmol/L (SD 1.5) for men and women, respectively. Additionally, 12 men (26%) and 23 women (32%) were found to have an elevated total or LDL-cholesterol. All outcome measures extracted purely from automated process are presented in table 1.

**Table 1:** Proposed outcome measures extracted from primary care computer records using an automated extraction at the end of the study period

Patient outcome measures	Male (n=46)	Female (n=72)
Age (years)	58 (9.0)	56 (7.5)
Total cholesterol (mmol/L)	5.8 (1.5)	6.4 (1.4)
LDL cholesterol (mmol/L)	3.6 (1.3)	3.8 (1.5)
Number with latest TC ≥7.5 mmol/L or LDL-C ≥4.9 mmol/L (%)	12 (26)	23 (32)
Number with repeat cholesterol test within 6 months after study start date (%)	41 (89)	60 (83)
Number of diagnosed with secondary cause* within 6 months after study start date (%)	5 (11)	5 (7)
Number with any family history of coronary heart disease assessed within 6 months after study start date (%)	14 (30)	31 (43)
Number with any complete family history† of coronary heart disease assessed within 6 months after study start date (%)	1 (2)	6 (8)
Number with TSH assessed within 6 months after study start date‡ (%)	14 (30)	17 (23)
Number with HbA1c assessed within 6 months after study start date§ (%)	6 (13)	17 (24)
Number with serum creatinine assessed within 6 months after study start date¶ (%)	19 (41)	29 (40)
Number with bilirubin, ALP or gamma GT assessed within 6 months after study start date** (%)	19 (41)	22 (31)
Number with arcus senilis or xanthalasma diagnosed within 6 months after study start date (%)	0 (0)	0 (0)
Number of prescribed any statins within 6 months after study start date (%)	14 (30)	13 (18)
Number of prescribed high-potency statins†† within 6 months after study start date (%)	4 (9)	2 (3)
Number of given dietary or weight management advice within 6 months after study start date (%)	27 (59)	33 (46)
Number of given smoking cessation advice within 6 months after study start date (%)	19 (41)	15 (21)

Variables are means and SDs unless otherwise specified.

*Diabetes, hypothyroidism, chronic kidney disease, liver disease.

†Complete family history is defined when age, condition and degree of relation to patient is documented.

‡Proxy for secondary investigations for hypothyroidism.

§Proxy for secondary investigations for diabetes.

¶Proxy for secondary investigations for chronic kidney disease.

**Proxy for secondary investigations for liver disease.

††Simvastatin ≥80 mg/day, atorvastatin ≥20 mg/day, rosuvastatin ≥10 mg/day.

ALP, alkaline phosphatase; GT, glutamyl transpeptidase; LDL, low density lipoprotein; TC, total cholesterol; TSH, thyroid stimulating hormone.

Considering other measures, manual extraction only showed a small absolute increase in number of patients diagnosed with a secondary causes of hypercholesterolaemia (men: +2%; women +3%), statin prescribing (men: +1%; women +4%), and in smoking cessation advice given (men: +2%; women: +8%). The low level of family history recording, particularly when identifying a ‘complete family history’, was only slightly improved by an absolute increase of 5% for both men and women (+2 men; +4 women) when manually extracting free text in the GP EHRs. However, in manual extraction, GPs arranged and documented more investigations for secondary causes (thyroid stimulating hormone: +30% for men; +36% for women; HbA1c: +18% for men, +33% for women; serum creatinine: +55% for men, +56% for women; liver function test: +52% for men, +58% for women), and dietary or weight management advice given (men: +12%; women: +20%). In automated computer data extraction, clinical features of FH (arcus senilis or xanthalasma) were not recorded, while in manual search, 7% (1 man; 7 women) of all participants had arcus senilis or tendon xanthalasma documented in free text.

## Discussion

This study showed that it is feasible to engage and recruit patients with raised cholesterol for more systematic identification of familial hypercholesterolaemia in primary care, both through opportunistic GP contact and by postal invitation. Although postal invitation resulted in more eligible patients being recruited than opportunistic invitation, the latter had double the response rate. This suggests both strategies should be adopted for patient recruitment in any future study. While only a feasibility study, new cases of definite FH were identified in the current work who are now on appropriate treatment and management pathways to reduce their previously elevated cardiovascular risk.

By using automated GP computer searches, we were able to capture relevant outcome measures for a proposed trial on lipid testing, statin prescribing and possible secondary causes of hypercholesterolaemia, without burdening patients and clinicians with data collection forms. This could potentially improve collating such outcome measures in this and other studies.

### Strengths and limitations

The majority of practices recruited to this study (four out of six) were from socially disadvantaged inner city populations where prior identification rates of FH were likely to be lower. Although one affluent rural practice accounted for nearly half the recruited participants, the approaches used successfully recruited patients from practices with underserved populations. In addition, successful extraction of outcome data from GP health records was achieved from 95% (118) of recruited patients using automated searches.

Owing to the pragmatic nature of this study, some patients may have received the recruitment packs twice (opportunistic and systematic). However, for ethical reasons, we could not identify patients given recruitment packs opportunistically, and did not consent to participate in the study. In terms of identifying eligible patients, we recognised that eligibility criteria did not consider the effects of statin treatment. There were individuals who had cholesterol levels below the 7.5 mmol/L threshold who were already on statins at the time of recording highest cholesterol level in the electronic medical records. If these treatment effects were considered,^16^ there may be more eligible patients than we previously thought. Additionally, recruitment of eligible patients to the study may have been influenced by healthcare policy and a gender bias in recruitment. The National Health Service vascular check programme screens^17^ offers CVD risk assessment for age range of 40–75 years and previous evidence^18^ have shown that women are more likely to join a general cardiovascular disease screening programme than men. Finally, the follow-up period of 6–12 months may have been too short for the outcome of specialist referral to be fed back into a patient's GP records.

### Clinical implications

Patients with definite FH have been identified from this study and are now benefiting from treatment to reduce their greatly elevated risk of premature CHD. Further, this allows for cascade screening of additional family members from definite cases of FH in secondary care, an approach that is known to be cost-effective.^19–22^

### Future research

This intervention, to more systematically identify patients with familial hypercholesterolaemia, is feasible to implement in GP, and facilitates successful recruitment of patients with raised cholesterol for appropriate assessment and referral to secondary care. Opportunistic recruitment may be improved in future studies by researchers more actively engaging with GP administrative and clinical staff, and by seeking to enhance effective implementation strategies,^23^ such as ensuring that study packs are available in all consultation rooms and repeated in-practice audit and feedback on the benefits of FH identification. To improve future implementation, we are currently assessing the post-study changes in practitioner behaviour on relevant clinical outcomes, such as family history and diagnosis of secondary causes, as well as the qualitative experience of those patients and healthcare professionals who participated in this study, including patients who declined follow-up.^24^

Using current (Simon-Broome) criteria, a large proportion of patients with possible FH are identified but not confirmed to have FH on specialist assessment. More resource-effective approaches to identify FH need to be developed.^25–28^ For instance, our recently developed approach (Familial Hypercholesterolaemia Case Acertainment Tool)^27^ from routinely available data, held in primary care EHRs, takes into account patients already on statins, secondary causes of raised cholesterol, triglycerides and premature CHD when identifying patients who may have FH.

The parameters derived in this study on eligibility, recruitment and diagnosis will directly inform a future cluster RCT in primary care. In any future trial, diagnosis of FH should be based on both clinical assessment and genetic testing. For a future trial in a large number of GPs, manual computer data extraction would likely not be feasible. Extraction of pseudoanonymised data from EHRs demonstrated in this and other related studies^29^ can rapidly capture key trial outcome measures without burdening patients, for example, with forms seeking detailed information which may reduce response rates.^30^ Nevertheless, coded (automated) data extraction needs to be improved, for example, by incorporating computer templates to collate process data and outcomes of referral. Other outcomes measures with low levels of recording, or not routinely assessed in primary care, such as detailed family histories and physical signs of FH (xanthalasma and arcus senilus), need to be supplemented by additional clinician training and facilitated data capture using validated questionnaires. Any future robust trial will need to adopt a cluster-randomised design with a follow-up duration of at least 12 months. The trial sample size should also take account of patients already on statins with pre-treatment cholesterol above the threshold.
